# Perceived Peer Integration, Parental Control, and Autonomy Support: Differential Effects on Test Anxiety during the Transition to Secondary School for Girls and Boys

**DOI:** 10.1007/s10964-024-02053-z

**Published:** 2024-07-17

**Authors:** Paulina Feige, Rainer Watermann

**Affiliations:** Department of Education and Psychology, Freie Universität Berlin, Berlin, Germany

**Keywords:** Test anxiety, School transition, Gender, Peer integration, Parental control, Parental autonomy support

## Abstract

Although previous research has investigated the impact of parents and peers on test anxiety in secondary or tertiary education, little is known about younger students, especially during the transition to secondary school. Additionally, it is unclear whether these social factors affect girls’ and boys’ test anxiety differently. Therefore, the current study examined the role of perceived peer integration into the new class context, perceived parental control, and autonomy support on girls’ and boys’ test anxiety (worry and emotionality) during the transition to secondary school. Data from 1770 students (*M*_*age*_ = 10.47, *SD* = 0.56; 51% females) were analyzed before (4th grade) and after the transition (5th grade) using a multigroup (girls vs. boys) structural equation model. Both facets of test anxiety decreased from 4th to 5th grade. Perceived peer integration into the new class was only relevant for girls’ test anxiety, while parental control predicted post-transition test anxiety for boys. The results suggest that the perceived social environment is an important factor in helping students cope with the demands of the transition to secondary school.

## Introduction

Test anxiety is considered a situation-specific personality trait triggered by evaluative stimuli (Zeidner & Matthews, 2003). In research literature, it is conceptualized as a multi-dimensional construct, most commonly based on facets of worry and emotionality. Worry refers to the cognitive component, including task-irrelevant thinking and deprecatory thoughts, while emotionality refers to increased physiological arousal, such as nervousness and tension. Both components are thought to reduce task-focused attention and impair performance, highlighting their importance for students’ academic success (Liebert & Morris, 1967). While research has demonstrated the impact of peers and parents for upper secondary or university students, less is known about younger children, particularly during the transition to secondary school. Additionally, there is little research on whether these social factors impact girls’ and boys’ test anxiety differently. Identifying predictors of test anxiety is critical, as it can limit children’s long-term academic development and well-being (Robson et al., 2023; von der Embse et al., 2018). Understanding these predictors during the transition period may be relevant for interventions targeting test anxiety. The aim of the present study is therefore to examine the impact of peers and parents on girls’ and boys’ test anxiety during the transition to secondary school.

### Secondary School Transition and Test Anxiety

“Surviving the Junior High School Transition”—the title of a transition study (Lord et al., 1994)—emphasizes the significance of the secondary school transition in a child’s life. The transition is marked by increased performance demands and major changes in classroom characteristics (Sirsch, 2003). In several European countries, such as Germany, Austria, Croatia, and Portugal, this transition occurs after 4th grade (Coelho et al., 2020; for an overview of European educational systems, see also Hoerner et al., 2015). It coincides with the period when children begin to incorporate evaluative feedback into their self-perceptions. Experiences of failure during this period can lead to a long-term fear of failure (Wigfield & Eccles, 1989). Research indicates that students who transition earlier (e.g., after the 5th grade compared to after the 6th grade), tend to develop more school-related anxiety in the long term (Grills-Taquechel et al., 2010).

While previous research about school transitions mostly examined the post-transition period, little is known about the importance of the pre-transition period for the development of test anxiety. Educational assessments in late elementary school can be considered *critical moments*, as they are highly relevant for students’ future educational careers (Flitcroft et al., 2017). For instance, in the German school system, a recommendation for the type of secondary school is made based on grades obtained in the last period of elementary school. Because studies indicate that perceived test importance fosters test anxiety (Eklöf & Nyroos, 2013; von der Embse et al., 2018), the time before the school transition, characterized by an increased emphasis on school grades, may be relevant for the development of test anxiety. In line with this, studies suggest that various types of anxiety are highest before the transition and decrease afterward (Symonds & Galton, 2014). Since school-related evaluative anxiety develops as a function of the respective school type and school performance (Valtin & Wagner, 2004), another possible explanation is that low-performing children in particular benefit from the transition, as their new social group tends to have similar levels of achievement (big-fish-little-pond effect, Marsh & Parker, 1984). To date, there is a lack of studies exploring the mean values and stability of test anxiety during the transition from elementary to secondary school in the context of early transition (after the 4th grade).

After the transition, children are confronted with new academic demands and school subjects, an increased emphasis on grading and social competition, as well as a new social learning environment with new classmates and a changing school size (Lohaus et al., 2004; Valtin & Wagner, 2004). Adapting to these academic changes and renegotiating their status in their new peer group requires a great deal of psychological and behavioral adjustment (Kingery et al., 2011; Langenkamp, 2010). On the one hand, these challenges provide children with the opportunity to learn and develop the skills they need for a successful academic career (Skinner & Raine, 2023). Thus, most students seem to adjust to their new environment without serious difficulties or even experience the transition as something positive (Lohaus et al., 2004; Sirsch, 2003). On the other hand, when the demands exceed their resources, children may struggle with these changes and experience declines in their developmental trajectories, such as their motivational orientation or self-confidence (Grolnick et al., 2000). Therefore, it might be worthwhile to take a differentiated perspective on children’s initial conditions and resources in late elementary school years that facilitate psychological adjustment to the new school context (Sirsch, 2003). In this context, the motivational theory of coping assumes that children’s interpersonal resources, such as parental or peer support are a crucial factor (Skinner & Wellborn, 1997). As previous research mostly examined the effects of peer (Hoferichter & Raufelder, 2015; Song et al., 2015) or parental support (Bouffard & Labranche, 2022; Song et al., 2015) on test anxiety from the 7th grade, empirical evidence on the predictors of test anxiety at the time of early transition (after the 4th grade) is generally lacking. A detailed understanding of the predictors in this particular age group might be beneficial to identify critical phases and antecedents of test anxiety development.

### Role of Peers

Considering that children spend a large part of their daytime at school, it does not seem surprising that peer relationships in their new class context are highly relevant for children’s psychological well-being and school adjustment. For instance, studies indicate the importance of the peer group for students’ academic achievement (Sebanc et al., 2016), school involvement (Kingery et al., 2011), and test and manifest anxiety in secondary school (Grills & Ollendick, 2002; Hoferichter & Raufelder, 2015; Rubin et al., 2015). Since it fulfills the basic need for relatedness, feeling accepted by the new peer group is considered a particular resource in dealing with life challenges such as the school transition (Langenkamp, 2010; Zimmer-Gembeck et al., 2023). Accordingly, even mild stressors can trigger anxiety and lead to maladaptive outcomes in children who feel threatened in their basic need for relatedness (Skinner & Wellborn, 1997).

At the age of school transition, relationships with peers become increasingly important (Bokhorst et al., 2010; Zimmer-Gembeck et al., 2023). However, the transition is accompanied by drastic changes in children’s social environment: Since former classmates often attend different schools, the children have to deal with new classmates and a new social reference group with a greater emphasis on social comparison (Bouffard & Labranche, 2022; Kingery et al., 2011). Hence, friendships in the new class are one of the key factors contributing to children’s sense of well-being (Curson et al., 2019). In contrast, a possible mismatch between the need for peer acceptance and actual peer integration after the transition may be particularly related to adjustment difficulties (Kingery et al., 2011). To date, the link between peer relationships and test anxiety during the transition to secondary school has received little attention so far. One study found a negative link between peer support and test anxiety after the transition (7th grade) to secondary school in a Korean sample (Song et al., 2015). However, this finding can only be applied to a limited extent to Western cultures due to cultural differences (Robson et al., 2023; Song et al., 2015). In addition, the time of the transition differs: While children change school after 4th grade in some European states, the transition in Korea happened after the 6th grade (Song et al., 2015). This is particularly worth mentioning, given the different development phases children may experience at this time. Thus, some research findings suggest that students who make the transition earlier may experience more pronounced difficulties than their older peers because their emotional resources may not yet be sufficient to deal with these changes (Grills-Taquechel et al., 2010). Therefore, it might be important to examine the factors that contribute to a successful transition in this particular age group.

### Role of Parents

During stressful life events, the family plays an important role as the primary source of comfort and support. For example, when a child is dealing with school demands like bad grades, parental reactions can determine what resources are available to regulate the event. Parental disciplinary measures, such as scolding or blaming, can further exacerbate the difficult situation and make maladaptive academic outcomes more likely (Skinner & Wellborn, 1997; Zimmer-Gembeck & Skinner, 2016). Despite the well-established assumption that parents have a significant impact on their children’s academic beliefs and behaviors, relatively little attention has been paid to the specific role they can play in facilitating their children’s successful academic adjustment during the transition to secondary school (Bouffard & Labranche, 2022).

In the tradition of socialization research, parental discipline practices are often classified as *parental control* and *parental autonomy support* (Ryan & Deci, 2000). Parental control is characterized by pressure and threats of punishment and thus appears to reduce children’s confidence in their ability to handle difficult situations on their own (Wigfield et al., 2015). There is ample empirical evidence that parental control is associated with anxiety (Ballash et al., 2006; Duchesne & Ratelle, 2010; Luis et al., 2008). Thus, test anxiety is thought to originate from negative parental reactions to performance in evaluative settings (Putwain et al., 2010). In secondary school (Ringeisen & Raufelder, 2015; Ritchwood et al., 2015) or college students (Putwain et al., 2010, Shadach & Ganor-Miller, 2013), parental control was found to be related to test anxiety, especially to cognitive test anxiety (Putwain et al., 2010; Shadach & Ganor-Miller, 2013). Research on younger children, especially at the time of the secondary school transition, is lacking. As previous findings are mostly based on cross-sectional studies (e.g., Putwain et al., 2010; Ringeisen & Raufelder, 2015), longitudinal effects remain unexplored.

In contrast to parental control, research on parental autonomy support differs in its conceptualizations. Some studies measure autonomy support as the dimensionally opposite pole of parental control (e.g., Grolnick et al., 2000). Recent conceptualizations of parental autonomy support encompass more than the absence of control, including aspects such as acknowledging the perspectives of the children, providing explanations, and offering choices whenever possible (McCurdy, et al., 2020; Zimmer-Gembeck & Skinner, 2016). In this way, children learn how to handle difficult situations and take responsibility for themselves (Spinrad et al., 2023). Studies based on this conceptualization suggest positive associations with academic adjustment and psychological well-being (for an overview, see Vasquez et al., 2016). Transferred to the time of transition, it is assumed that children who experience themselves as more autonomous also develop adaptive strategies to deal with the new challenges (Grolnick et al., 2000). To date, there are no studies on the relation between parental autonomy support and test anxiety during transition to secondary school. Examining this relation may deepen the understanding of specific parental behaviors that affect test anxiety.

### Gender Differences

While there is compelling evidence that girls report more test anxiety (Putwain & Daly, 2014; Ritchwood et al., 2015; Robson et al., 2023), more social support from parents and friends (Bokhorst et al., 2010; Colarossi & Eccles, 2003), and less parental control (Duchesne & Ratelle, 2010), there is little research on whether social factors have a sex-differential effect on test anxiety. In general, research findings indicate that girls are more concerned about their dyadic relationships and peer evaluation, report more stress in their peer group, and rely more on social support to cope with stressors (Rose & Rudolph, 2006). Moreover, girls tend to establish intimate friendships at an earlier stage of adolescence than boys and are more likely to experience higher levels of intimacy in these relationships. This intimacy may render girls more vulnerable when their relationships are disrupted. Indeed, girls exhibit greater levels of anxiety than boys in response to stressors in their interpersonal relationships (Kingery et al., 2011; Rose & Rudolph, 2006). As a possible consequence, the negative effects of transitions are thought to be greater for girls than for boys (Anderson et al., 2000). While these results provide evidence that girls’ well-being is generally more affected by social factors, particularly their peer relations, empirical literature examining gender-differential effects in test anxiety is limited. Two studies found no gender differences in the relation between peer and parental support and test anxiety (Hoferichter & Raufelder, 2015; Song et al., 2015). Since both studies examined gender differences in adolescents from 7th grade, little is known about gender differences in younger children, especially at the time of early school transition (after the 4th grade) as it occurs in most German federal states. Understanding whether differential factors contribute to boys’ and girls’ test anxiety could be highly relevant for intervention programs.

## Current Study

To date, there has been a lack of studies on test anxiety that cover the period of early school transition (after the 4th grade). While there is ample evidence that girls report more test anxiety than boys, less is known about differential predictors of test anxiety in boys and girls. Based on a one-year longitudinal design, the first aim of this study was to capture test anxiety before and after the transition in terms of mean values and rank orders. Based on the motivational theory of coping, the second aim was to examine the longitudinal impact of peers and parents on girls’ and boys’ test anxiety at the time of school transition. Since peer relationships are a crucial resource and thus facilitate the adjustment to the new context, it was expected that peer integration is negatively related to test anxiety after the transition (Hypothesis 1). Framed by the motivational theory of coping, it was further assumed that parental control might add stress to the challenging situation of school transition, making maladaptive outcomes more likely, while parental autonomy support might facilitate the adjustment process. Therefore, it was expected that pre-transition parental control is positively related to test anxiety (Hypothesis 2), and parental autonomy support is negatively related to test anxiety after the transition (Hypothesis 3). Since there is less evidence on differential predictors for girls and boys, gender differences were tested exploratively. Due to findings that school-related evaluative anxiety develops as a function of the respective school type and school performance, both variables were included as control variables. To consider the initial level of test anxiety when moving to secondary school, it was additionally controlled for test anxiety in the last year of elementary school.

## Method

### Sample and Procedure

The data used in the present study originate from the longitudinal project *Trends in International Mathematics and Science Study - Transition Study* (TIMSS; Becker et al., 2010). A variety of motivational and psychosocial variables before and after the transition to secondary school were assessed in the study. The data collection began in November 2006 and ended in October 2008. For the current study, data from two measurement points in 4th grade (before transition, April/May 2007) and 5th grade (after transition, February/March 2008) were used. The data were collected by standardized achievement tests and student questionnaires. Participants were drawn from 247 elementary schools in 13 states in Germany (excluding Berlin, Brandenburg, and Mecklenburg-Vorpommern). All students who participated in at least one measurement point after the transition were included. To ensure that the sample included only children who transferred to secondary school after 4th grade, all students who indicated *elementary school* as their school type in 5th grade or who had missing values for school type were excluded. For the present investigation, data of 1770 students (*M*_*age*_ = 10.47, *SD* = 0.56; 51% females) were used. As another study has shown, the longitudinal sample is positively selected. Compared to the dropouts, the remaining participants had better grades in German and math, and a higher social status (Zhang et al., 2023). This should be considered when assessing the external validity of the study results.

### Measures

#### Test anxiety

Two facets of test anxiety were measured: worry (4th grade: α = 0.77, 5th grade: α = 0.71) and emotionality (4th grade: α = 0.86, 5th grade: α = 0.85) before (4th grade) and after (5th grade) the transition to secondary school. Due to unclear loading patterns, two items were excluded from the analyses. The final worry scale (Helmke, 1992) consisted of three items (e.g., “I thought about all the things I can’t do”). Emotionality (Hodapp et al., 1982) was measured by four items (e.g., “I was so nervous that I could hardly work anymore”). Both scales were measured on a 4-point Likert scale from *not true at all* (1) to *totally true* (4).

#### Peer integration

Peer integration into the new class context (α = 0.68) was measured after the transition to secondary school (5th grade) by three items: “I have a lot of friends in my class“; “I meet with my classmates after school“; “It is hard to make friends at our school“. The first two items were recoded, so that a higher score was also associated with higher peer integration. The children could answer on a 4-point Likert scale from *not true at all* (1) to *totally true* (4).

#### Parental control and autonomy support

Both parental behaviors were assessed using a questionnaire developed by Wild (1999) and Lorenz & Wild (2007). Parental control (α = 0.79) was measured in 4th grade. After students were asked: “You don’t always succeed in getting a good grade in school. When you get a bad grade, what happens at home?”, they had to answer four items (e.g., “My parents scold me and demand that I learn more“). Parental autonomy support (α = 0.68) was also measured in 4th grade by four items (e.g., “My parents don’t tell me right away what I should do, but listen calmly to how I want to deal with the situation myself“). Both scales were measured on a 4-point Likert scale from *not true at all* (1) to *totally true* (4).

#### Type of school after transition

The type of school after the transition was reported by parents at the end of elementary school (4th grade). The variable was included as a dichotomous variable with the two options *other type of school* (0) and *gymnasium*^1^(1). Accordingly, 56.13% of the children attended the gymnasium after the school transition.

#### Standardized achievement tests

Standardized achievement tests in German and math were administered at the end of 4th grade (for a detailed description see Becker et al., 2010). The math test included a total of 179 multiple-choice and short-answer questions (α = 0.83) on arithmetic (52%), geometry/measurement (34%), and data (15%). The German achievement test included a total of 446 items (WLE reliability, r = 0.81) on the skill areas of reading, listening, language use, and spelling. Due to the multi-matrix sampling, not all items were presented to all students (Becker et al., 2010).

### Statistical Analysis

Scale scores were used to compute descriptive statistics and *t*-tests. To validate the theoretical assumption of worry and emotionality as two facets of test anxiety, confirmatory factor analyses were conducted for the measures of test anxiety. Furthermore, in a stepwise approach (Brown, 2015), multiple-group confirmatory factor analyses were performed to examine the invariance of all variables across groups (boys and girls). Because test anxiety was included at two measurement time points, both facets were tested for measurement invariance across time. To evaluate measurement invariance, the models were compared based on the change in CFI (∆CFI). Accordingly, the change should be smaller or equal to −0.01 to assume measurement invariance (Cheung & Rensvold, 2002).

**Fig. 1:**
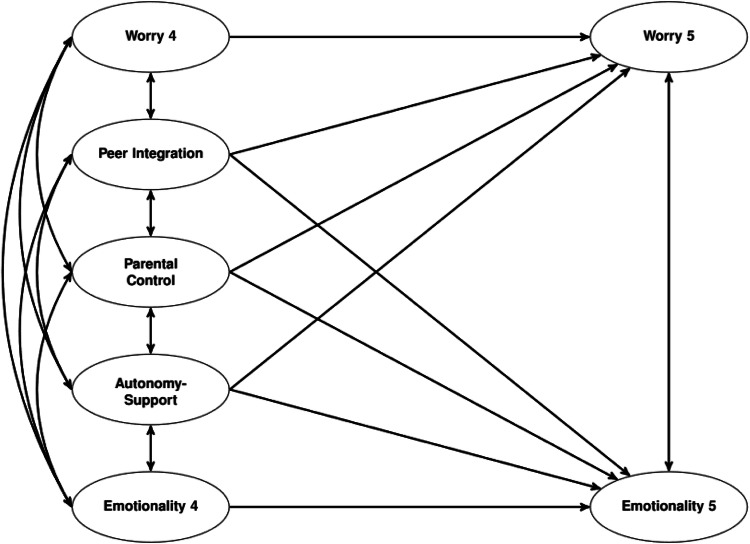
Structural equation model. Numbers indicate grade levels. In addition, it was controlled for standardized math and German achievement scores and school type after transition

To test the hypotheses, a structural-equation model was applied (Fig. 1), using M*plus* 8.7 (Muthén & Muthén, 1998–2021) with maximum likelihood estimation with robust standard errors. Model fit was estimated by the following fit indices, as recommended by Hu and Bentler (1999): Chi-Square Test of Model Fit (χ²/df), Root Mean Square Error of Approximation (RMSEA), and its 90% Confidence Interval, Comparative Fit Index (CFI), Tucker-Lewis Index (TLI) and Standardized Root Mean Square Residuals (SRMR). Cutoff values close to 0.06 for RMSEA, 0.95 for CFI, and 0.08 for SRMR are considered indicators of good model fit (Hu & Bentler, 1999). The model was tested as a two-group model, each for boys and girls. Correlations between residuals of identical items over time were allowed. To test for possible gender differences in the model paths, *χ²-*difference tests with Satorra–Bentler scaling correction were applied.

Full-information maximum likelihood (FIML) was used to address missing data (Enders & Bandalos, 2001). In the present study, the percentage of missing values ranged across the measurement points from 5.3% to 9.10% for worry and emotionality. The percentage of missing values was 5.59% for parental control and autonomy support and 7.79% for peer integration.

## Results

Confirmatory factor analyses confirmed the two-factorial structure of test anxiety (worry and emotionality) for both measurement time points (see Table 3, supplementary material).

### Invariance Testing Across Time and Groups

Before conducting further analyses, multiple-group confirmatory factor analyses were performed with all measures to examine invariance across groups and, for worry and emotionality, across time (Table 4, supplementary material). Since the change in CFI did not exceed the threshold of 0.01, longitudinal scalar invariance was confirmed for worry and emotionality, meaning that the intercepts were equal over time. Except for worry, the results also support scalar invariance between girls and boys. With regard to worry, partial scalar gender invariance was supported. Based on the results of the invariance testing, the final model (Fig. 1) assumed equal factor loadings across groups and -for worry and emotionality- across both measurement points. With the exception of the non-invariant item of worry, indicator intercepts were hold equal across groups and time. Model fit was good, χ² (653) = 1154.036, *p* < 0.001; CFI = 0.961; TLI = 0.955; RMSEA = 0.029, 90% CI (0.027; 0.032); SRMR = 0.043.

### Descriptive Results, Mean Differences, and Bivariate Correlations

Mean values, standard deviations, and bivariate correlations of all variables examined can be found in Table 1. To test mean differences between boys and girls, a multiple indicators multiple causes model (MIMIC, Wells, 2021) was used to account for the non-invariant indicator of worry. Girls and boys did not differ in their worry in 4th grade, β = 0.034, *p* = 0.430. With boys as the reference group, mean comparisons showed that girls reported significantly more worry in 5th grade, β = 0.084, *p* =0.025. Girls reported significantly more emotionality in 4th, β = 0.088, *p* = 0.004; and 5th grade, β = 0.138, *p* < 0.001; and significantly more peer integration after the transition, β = 0.104, *p* = 0.001. Boys reported significantly more parental control, β = −0.195, *p* < 0.001. No gender difference in parental autonomy support was found, β = 0.067, *p* = 0.070.

**Table 1:** Means, standard deviations, and bivariate correlations for girls (above the diagonal) and boys (below the diagonal)

Variable^a^	Girls		Boys								
	*M*	*SD*	*M*	*SD*	1	2	3	4	5	6	7
1 Worry4	2.12	0.90	2.16	0.88		0.38^***^	0.72^***^	0.31^***^	−0.12^**^	0.37^***^	0.17^***^
2 Worry5	1.93	0.80	1.90	0.75	0.30^***^		0.34^***^	0.65^***^	−0.20^***^	0.25^***^	0.08^*^
3 Emo4	1.89	0.85	1.77	0.79	0.70^***^	0.25^***^		0.35^***^	−0.06	0.36^***^	0.12^*^
4 Emo5	1.79	0.76	1.62	0.69	0.22^***^	0.65^***^	0.27^***^		−0.13^***^	0.16^***^	0.11^**^
5 PI	3.58	0.51	3.46	0.59	−0.10^**^	−0.08^**^	−0.12^***^	−0.12^***^		−0.13^***^	0.05
6 PC	1.53	0.65	1.72	0.77	0.40^***^	0.25^***^	0.42^***^	0.17^***^	−0.12^***^		
7 PS	3.01	0.70	2.94	0.76	0.17^***^	0.06	0.12^**^	0.07	0.08^*^	0.07^*^	

Descriptive statistics and correlations are based on sum scores

*Emo* emotionality, *PI* peer integration, *PC* parental control, *PS* parental autonomy support

^*^p < 0.05. ^**^p < 0.01. ^***^p < 0.001

^a^Numbers indicate grade levels

The bivariate correlations can be found in Table 1. Peer integration was negatively related to worry in 4th and 5th grade and emotionality in 5th grade for girls. Peer integration was negatively related to boys’ worry and emotionality at both measurement points. Parental control was positively correlated with girls’ and boys’ worry and emotionality at both measurement points. Parental autonomy support was positively related to worry (girls: 4th & 5th grade, boys: 4th grade) and emotionality (girls: 4th & 5th grade; boys: 4th grade).

### Test Anxiety Before and After the Transition

To explore test anxiety before and after the transition, the mean values (Table 1), which describe the average means at the two measurement points, and the autoregressive paths (Table 2), which describe the stability of individual differences in worry and emotionality over time, were examined. Descriptively, both test anxiety facets decreased for boys and girls after school transition, indicating that test anxiety was highest in the last year of elementary school. Controlling for the other predictors, all autoregressive paths were significant, indicating that the assessment of worry/emotionality in 4th grade predicted the assessment of worry/emotionality in 5th grade, respectively. The effect sizes indicate small to moderate stability in individual rank orders across the transition.

**Table 2:** Unstandardized and standardized path coefficients on girls’ and boys’ worry and emotionality

Path^a^	Girls			Boys			
	*b*	*SE(b)*	β	*b*	*SE(b)*	β	Δχ^2b^
Hypothesis 1							
PI → WOR5	−0.350^***^	0.081	−0.237^***^	−0.050	0.050	−0.047	16.105^***^
PI → EMO5	−0.211^***^	0.058	−0.179^***^	−0.047	0.042	−0.054	6.191^*^
Hypothesis 2							
PC → WOR5	0.120	0.079	0.100	0.167^**^	0.061	0.181^**^	0.250
PC → EMO5	0.016	0.057	0.017	0.063	0.044	0.085	0.490
Hypothesis 3							
PS → WOR5	0.027	0.053	0.024	−0.012	0.048	−0.013	0.318
PS → EMO5	0.072	0.040	0.080	0.021	0.035	0.027	0.340
Stability coefficients							
WOR4 → WOR5	0.312^***^	0.050	0.361^***^	0.231^***^	0.055	0.275^***^	–
EMO4 → EMO5	0.305^***^	0.045	0.358^***^	0.265^***^	0.050	0.307^***^	–
Covariates							
ST → WOR5	0.096	0.065	0.068	0.142^*^	0.056	0.112^*^	–
ST → EMO5	0.144^**^	0.051	0.128^**^	0.131^**^	0.041	0.128^**^	–
MAT → WOR5	−0.007	0.004	−0.090	0.001	0.004	0.015	–
MAT → EMO5	−0.006^*^	0.003	−0.108^*^	0.000	0.003	0.003	–
GAT → WOR5	0.004	0.004	0.048	−0.003	0.004	−0.049	–
GAT → EMO5	−0.001	0.003	−0.015	−0.001	0.003	−0.028	–

*WOR* worry, *EMO* emotionality, *PI* peer integration, *PC* parental control, *PS* parental autonomy support, *ST* school type after transition (0 = academic track, 1 = other), *MAT* math achievement test, *GAT* German achievement test

^*^p < 0.05. ^**^p < 0.01. ^***^p < 0.001

^a^Arrow indicates direction of causation

^b^df = 1

### Peer Integration and Test Anxiety

The model results can be seen in Table 2. Note that the effects on girls’ and boys’ test anxiety in 5th grade were estimated while controlling for the test anxiety in 4th grade. Thus, all effects can be interpreted as effects on test anxiety in 5th grade, independently of the child’s prior test anxiety in elementary school. For girls, worry and emotionality in 5th grade were negatively predicted by peer integration into the new class context after transition. In line with Hypothesis 1, this suggests that girls who felt socially better integrated had less test anxiety in 5th grade. For boys, no effect of peer integration on both text anxiety facets in 5th grade was found. The results of the *χ²-*difference tests indicate significant differences between girls and boys. This indicates that peer integration predicts worry and emotionality after the transition significantly more strongly for girls than for boys.

### Parental Control and Test anxiety

For girls, parental control had no significant effect on both facets of test anxiety after the transition (5th grade). For boys, there was an effect of parental control on worry after school transition (5th), but no effect on emotionality. The results of the *χ²-*difference tests indicate that the difference between boys and girls was not significant.

### Parental Autonomy Support and Test Anxiety

Contrary to Hypothesis 3, no effects of parental autonomy support on girls’ and boys’ worry and emotionality in 5th grade were found. The results of the *χ²-*difference tests indicate no significant gender differences.

## Discussion

For many children, the transition to secondary school is the first significant change in their academic life, requiring substantial psychological adjustment to the new school context. The current study aimed to explore test anxiety (worry and emotionality) before and after the transition and to investigate longitudinal effects of perceived peer integration, parental control, and parental autonomy support on worry and emotionality among girls and boys during this period. Following the motivational theory of coping (Skinner & Wellborn, 1997), it was assumed that social factors such as perceived peer integration and parental behavior can either support or hinder this adjustment process and therefore may be related to test anxiety. The findings suggest that both test anxiety facets were the highest before the transition. Independently of prior test anxiety in elementary school, peer integration into the new class context appeared to be only relevant for worry and emotionality among girls (Hypothesis 1). Parental control predicted post-transition worry only for boys (Hypothesis 2). Contrary to Hypothesis 3, no effects of parental autonomy support on girls’ and boys’ test anxiety in 5th grade were found.

### Gender Differences

Consistent with other study findings (Putwain & Daly, 2014; Ritchwood et al., 2015; Robson et al., 2023), girls reported higher levels of emotionality and worry in this study. This suggests that girls may be a risk group for developing test anxiety. It is generally assumed that different gender-specific socialization leads to girls showing more internalizing symptoms. For example, parents consider fear and avoidance to be less acceptable for boys, causing boys to learn different coping mechanisms than girls (McLean & Anderson, 2009). Consistent with other findings (Bokhorst, et al., 2010; Colarossi & Eccles, 2003), girls reported significantly more peer integration after the transition. In line with Duchesne & Ratelle (2010), it was found that boys perceive their parents as more controlling than girls. In contrast, no differences in parental autonomy support were found.

### Test Anxiety Before and After the Transition

Regarding the mean values of test anxiety, boys and girls reported the highest test anxiety before the transition. The autoregressive effects from 4th to 5th grade indicate a small to moderate rank order stability along the transition. The good news is that both test anxiety facets tended to decrease slightly after the transition. This may be related to the fact that the last period of elementary school is characterized by increased pressure to perform since this performance impacts the recommendation for the secondary school type. As study findings suggest that test anxiety increases during high-stakes exams, the time before transition might be crucial for the development of test anxiety (Robson et al., 2023; von der Embse et al., 2018). Another possible explanation is that low-performing children benefit from the transition, as their new social group tends to have similar levels of achievement (big-fish-little-pond effect, Marsh & Parker, 1984).

On average, the major classroom changes in secondary school were not associated with an increase in test anxiety for the students, supporting the thesis that academic challenges like the transition to secondary school can also be associated with learning opportunities and positive consequences (Sirsch, 2003). Alternatively, this result may also reflect general developmental processes, which are not necessarily attributable to the transition to the secondary level. Therefore, this assumption should be tested by follow-up studies, comparing trajectories in samples of students with early (after the 4th grade) and late (after the 6th grade) transition.

### Peer Integration and Test Anxiety

The results support Hypothesis 1 for girls only. Peer integration had a negative effect on worry and emotionality after the transition for girls. No effect was found for boys, and the difference between boys and girls was significant. For the sample of this study, peer integration in the new class was relevant for girls’, but not for boys’ test anxiety. In line with other studies, one can conclude that girls’ well-being is stronger affected by social acceptance (for an overview, see Rose & Rudolph, 2006). Thus, girls seemed to be more concerned about their social relationships, desired close relationships more, and were more likely to use social support to cope with stressors than boys. Hence, the transition period may be particularly challenging for girls’ well-being because of the uncertainty about social relationships in the new class context. Moreover, since both boys and girls tend to have same-sex friendships (Rose & Rudolph, 2006), different sex-typed peer relationship styles need to be considered in future research. Since girls tend to show more empathy and sensitivity to others’ distress, their friendships may provide greater support in challenging situations (Rose & Rudolph, 2006).

However, the findings cannot be generalized in the way that social aspects are not relevant for boys. Based on need theories (e.g., Maslow, 1943; Ryan & Deci, 2000) it can be assumed that boys also benefit from relatedness to others. Indeed, two studies (Hoferichter & Raufleder, 2015; Song et al., 2015) found a negative link between peer support and test anxiety for both boys and girls. Since both study samples were adolescents between 7th and 9th grade, and therefore older compared to the sample in this study, developmental aspects should be taken into account. For instance, girls may establish intimate friendships at an earlier stage than boys (Kingery et al., 2011). This could be examined by further studies, which trace the effects over time. Furthermore, friendships that exist outside of school may also be important (Bokhorst et al., 2010). In addition, students might benefit from close friendships differently than from classroom integration in general. Future studies should take this into account by shedding light on the quality of individual friendships and their impact on test anxiety.

### Parental Control and Test Anxiety

The results support Hypothesis 2 for boys. Consistent with prior work, it was assumed that parental control affects test anxiety (e.g., Putwain et al., 2010; Ringeisen & Raufelder, 2015). The results of this study suggest that negative parental feedback on academic performance during the transition to secondary school, a period characterized by an increased emphasis on graded performance, may particularly contribute to the development of boys’ test anxiety in secondary school. Viewed through the lenses of the motivational theory of coping (Skinner & Wellborn, 1997), it can be hypothesized that parental reactions counteract boys’ self-regulation and reduce their confidence to handle transition challenges on their own. It is important to recognize that the process of coping, which is intermediately described in the model, could not be considered in this study. Thus, it is assumed that parental control impairs children’s coping (e.g., self-pity, rumination), which in turn affects developmental outcomes, like children’s test anxiety (Skinner & Wellborn, 1997; Spinrad et al., 2023). This process should be investigated in follow-up studies.

In studies that did not specifically investigate gender disparities, parental control was found to be especially related to cognitive test anxiety (Putwain et al., 2010; Shadach & Ganor-Miller, 2013). Differentiating by child’s gender, the results of this study suggest that parental control was only related to boys’ cognitive test anxiety. On average, boys perceived their parents as more controlling compared to girls, which was linked to higher worries in test situations. By aggregating the scores for both genders, differential effects of parental control may have been masked in past studies. The findings of this study suggests that differentiation by gender in terms of factors influencing test anxiety might be useful.

### Parental Autonomy Support and Test Anxiety

The results do not support Hypothesis 3. Controlling for the other predictors, no effects of parental autonomy support on girls’ and boys’ post-transition test anxiety were found. The results of this study suggest that parental autonomy support plays a minor role in the development of test anxiety compared to parental control and peer integration. However, the descriptive results suggest significant bivariate relations between parental autonomy support and girls’ and boys’ test anxiety. These relations were positive, indicating that higher levels of parental autonomy support were related to higher levels of test anxiety. Generally, autonomy-supportive parental behaviors were thought to have positive effects on academic outcomes, like self-esteem, intrinsic motivation, and psychological well-being (Vasquez et al., 2016). While recent works (Bouffard & Labranche, 2022; Grolnick et al., 2000) mostly conceptualized parental autonomy support as a general parenting style, this study focused on autonomy support as a reaction to school grades. It can be assumed that the operationalization in this study also expresses the importance of good grades for parents. For instance, parents who place a lower priority on good grades may be less likely to bring this up. Conversely, parents discussing grades emphasize the importance of school performance. As students strive to meet their parents’ expectations, they may put pressure on themselves to perform well, which in turn may be associated with maladaptive outcomes, like test anxiety (Raufelder & Ringeisen, 2015).

### Strengths and Limitations

This study has several strengths and limitations. One strength of the present study is that it addresses the need for longitudinal research. While most studies only examined the period after the transition, this study covers the transition from the last grade in elementary school to the first grade in secondary school, allowing for an examination of test anxiety before and after the transition. The findings suggest that social factors before and after the transition may have an impact on test anxiety in secondary school, independently from standardized achievement scores and school type after the transition. Additionally, gender-specific patterns were analyzed, revealing a significant difference between boys and girls in terms of integration into new peer environments. This underscores the relevance of considering gender differences in future studies.

Despite these merits, this study has some limitations. First, parenting may change across the transition. It therefore remains unclear whether the perceived parental behavior in the last year of elementary school is responsible for test anxiety in the first year of secondary school. However, study results indicate a moderate to high stability in parenting behaviors over one year, suggesting that significant changes are unlikely between 4th and 5th grade (e.g., Loeber et al., 2000; bivariate correlations of repeated measures along the transition reported by Wang et al., 2023). Furthermore, the direction of influence is also debatable: parental behavior likely reacts to child behavior (Kerr & Stattin, 2003). Further research should take this into account by using research designs that can provide stronger causal evidence (e.g., cross-lagged panel). Moreover, this study assessed children’s perceived parental behavior. This seems reasonable, as it can be assumed that the child’s interpretation of parental behavior in particular is crucial for the development of test anxiety. When interpreting the findings, it must be kept in mind that actual parental behavior may differ. Furthermore, since the longitudinal sample was positively selected, it can be assumed that this study represents disproportionately high-achieving children. To minimize this potential bias, it was controlled for standardized achievement tests. Furthermore, multidimensional models of test anxiety include not only worry and emotionality but also facets such as lack of confidence or interference (Hodapp, 1995). This study focused on worry and emotionality, as Liebert and Morris’ (1967) two-factor model is the most widely used conceptualization. Cultural differences, such as varying societal norms and different parenting styles between Eastern and Western cultures (Robson et al., 2023), suggest that the findings of this study may have limited applicability to members of Eastern cultures.

## Conclusion

Despite previous research investigating the role of parents and peers in upper secondary school or university, little attention has been paid to younger students, especially during the transition to secondary school. Furthermore, little is known about whether parents and peers may have a differential impact on girls’ and boys’ test anxiety. By using a longitudinal design that includes 4th grade (before the transition) and 5th grade (after the transition), the present study examines the effects of perceived peer integration into the new class context, perceived parental control, and autonomy support on girls’ and boys’ test anxiety. The results support the idea that social factors play a role in the development of test anxiety during the school transition period and that there are differential predictors for girls and boys. According to the findings, test anxiety was highest on average before the transition and decreased slightly afterward. In line with this, the results suggest that boys whose parents were more controlling and girls who were less integrated into their new classes recovered less from the demands of the transition in terms of their test anxiety. The findings of this study extend the knowledge of the predictors in this particular age group of adolescents and provide practical implications for prevention and intervention programs.

## Supplementary Information

Supplementary Material

## Data Availability

The datasets analyzed during the current study are not publicly available but are available from the corresponding author on reasonable request.

## Footnotes

1 University Track

## References

[CR1] Anderson, L. W., Jacobs, J., Schramm, S., & Splittgerber, F. (2000). School transitions: Beginning of the end or a new beginning? *International Journal of Educational Research*, *33*(4), 325–339. 10.1016/S0883-0355(00)00020-3.

[CR2] Ballash, N., Leyfer, O., Buckley, A. F., & Woodruff-Borden, J. (2006). Parental control in the etiology of anxiety. *Clinical Child and Family Psychology Review*, *9*, 113–133. 10.1007/s10567-006-0007-z. 17089199 10.1007/s10567-006-0007-z

[CR65] Becker, M., Gresch, C., Baumert, J., Watermann, R., Schnitger, D., & Maaz, K. (2010). Durchführung, Daten und Methoden [Procedure, data and methods]. In K. Maaz, J. Baumert, C. Gresch & N. McElvany (Hrsg.), Der Übergang von der Grundschule in die weiterführende Schule – Leistungsgerechtigkeit und regionale, soziale und ethnisch-kulturelle Disparitäten (S. 107–121). BMBF.

[CR3] Bokhorst, C. L., Sumter, S. R., & Westenberg, P. M. (2010). Social support from parents, friends, classmates, and teachers in children and adolescents aged 9 to 18 years: Who is perceived as most supportive? *Social Development*, *19*(2), 417–426. 10.1111/j.1467-9507.2009.00540.x.

[CR4] Bouffard, T., & Labranche, A.-A. (2022). Profiles of parenting autonomy support and control: A person-centered approach in students’ adjustment to the transition to middle school. *The Journal of Early Adolescence*, *43*(7), 908–946. 10.1177/02724316221136039.

[CR5] Brown, T. A. (2015). *Confirmatory factor analysis for applied research* (2nd ed.). The Guilford Press.

[CR6] Cheung, G. W., & Rensvold, R. B. (2002). Evaluating goodness-of-fit indexes for testing measurement invariance. *Structural Equation Modeling*, *9*(2), 233–255. 10.1207/S15328007SEM0902_5.

[CR7] Coelho, V. A., Bear, G. G., & Brás, P. A. (2020). Multilevel analysis of the importance of school climate for the trajectories of students’ self-concept and self-esteem throughout the middle school transition. *Journal of Youth and Adolescence*, *49*, 1793–1804. 10.1007/s10964-020-01245-7. 32356038 10.1007/s10964-020-01245-7

[CR8] Colarossi, L. G., & Eccles, J. S. (2003). Differential effects of support providers on adolescents’ mental health. *Social Work Research*, *27*(1), 19–30. 10.1093/swr/27.1.19.

[CR9] Curson, S., Wilson-Smith, K., & Holliman, A. J. (2019). Exploring the experience of students making the transition from primary school to secondary school: An interpretative phenomenological analysis of the role of friendship and family support. *Psychology Teaching Review*, *25*(1), 30–41. 10.53841/bpsptr.2019.25.1.30.

[CR10] Duchesne, S., & Ratelle, C. F. (2010). Parental behaviors and adolescents’ achievement goals at the beginning of middle school: Emotional problems as potential mediators. *Journal of Educational Psychology*, *101*, 497–507.

[CR11] Eklöf, H., & Nyroos, M. (2013). Pupil perceptions of national tests in science: Perceived importance, invested effort, and test anxiety. *European Journal of Psychology of Education*, *28*, 497–510. 10.1007/s10212-012-0125-6.

[CR12] Enders, C. K., & Bandalos, D. L. (2001). The relative performance of full information maximum likelihood estimation for missing data in structural equation models. *Structural Equation Modeling*, *8*(3), 430–457.

[CR13] Flintcroft, D., Woods, K., & Putwain, D. W. (2017). Developing school practice in preparing students for high-stake examinations in English and Mathematics. *Educational and Child Psychology*, *34*(3), 7–19.

[CR14] Grills-Taquechel, A. E., Norton, P., & Ollendick, T. H. (2010). A longitudinal examination of factors predicting anxiety during the transition to middle school. *Anxiety Stress Coping*, *23*(5), 493–513. 10.1080/10615800903494127. 20711893 10.1080/10615800903494127 PMC2924763

[CR15] Grills, A. E., & Ollendick, T. H. (2002). Peer victimization, global self-worth, and anxiety in middle school children. *Journal of Clinical Child and Adolescent Psychology*, *31*(1), 59–68. 10.1207/153744202753441675. 11845651 10.1207/S15374424JCCP3101_08

[CR16] Grolnick, W., Kurowski, C. O., Dunlap, K. G., & Hevey, C. (2000). Parental resources and the transition to junior high. *Journal of Research on Adolescence*, *10*(4), 456–488. 10.1207/SJRA1004_05.

[CR17] Helmke, A. (1992). *Selbstvertrauen und schulische Leistungen* [Self-confidence and academic performance]. Hogrefe.

[CR18] Hodapp, V. (1995). The TAI-G: A multidimensional approach to the assessment of test anxiety. In C. Schwarzer & M. Zeidner (Eds.), *Stress, anxiety, and coping in academic settings (*pp. 95–130). Francke.

[CR19] Hodapp, V., Laux, L., & Spielberger, C. (1982). Theorie und Messung der emotionalen und kognitiven Komponente der Prüfungsangst [Theory and measurement of the emotional and cognitive components of test anxiety]. *Zeitschrift für Differentielle und Diagnostische Psychologie*, *3*, 169–184.

[CR20] Hoerner, W., Doebert, H., Reuter, L. R., Kopp, B. (Eds.). (2015). *Educational Systems in Europe, Global Education Systems*. Springer Cham. 10.1007/978-3-319-07473-3.

[CR21] Hoferichter, F., & Raufelder, D. (2015). Examining the role of social relationships in the association between neuroticism and test anxiety: Results from a study with German secondary school students. *Educational Psychology*, *35*(7), 851–868. 10.1080/01443410.2013.849326.

[CR22] Hu, L., & Bentler, P. M. (1999). Cutoff criteria for fit indexes in covariance structure analysis: Conventional criteria versus new alternatives. *Structural Equation Modeling: A Multidisciplinary Journal*, *6*(1), 1–55. 10.1080/10705519909540118.

[CR23] Kerr, M., & Stattin, H. (2003). Parenting of adolescents: Action or reaction? In A. C. Crouter & A. Booth (Eds.), *Children’s influence on family dynamics: The neglected side of family relationships* (pp. 121–151). Lawrence Erlbaum Associates Publishers.

[CR24] Kingery, N. J., Erdley, C. A., & Marshall, K. C. (2011). Peer acceptance and friendship as predictors of early adolescents’ adjustment across the middle school transition. *Merrill-Palmer Quarterly*, *57*(3), 215–243. 10.1353/mpq.2011.0012.

[CR25] Langenkamp, A. G. (2010). Academic vulnerability and resilience during the transition to high school: The role of social relationships and district context. *Sociology of Education*, *83*(1), 1–19. 10.1177/0038040709356563.

[CR26] Liebert, R. M., & Morris, L. W. (1967). Cognitive and emotional components of test anxiety: A distinction and some initial data. *Psychological Reports*, *20*, 975–978. 10.2466/pr0.1967.20.3.975. 6042522 10.2466/pr0.1967.20.3.975

[CR27] Loeber, R., Drinkwater, M., Yin, Y., Anderson, S. J., Schmidt, L. C., & Crawford, A. (2000). Stability of family interaction from ages 6 to 18. *Journal of Abnormal Child Psychology*, *28*(4), 353–369. 10.1023/A:1005169026208. 10949960 10.1023/a:1005169026208

[CR28] Lohaus, A., Elben, C. E., Ball, J., & Klein-Hessling, J. (2004). School transition from elementary to secondary school: Changes in psychological adjustment. *Educational Psychology*, *24*(2), 161–173. 10.1080/0144341032000160128.

[CR29] Lord, S. E., Eccles, J. S., & McCarthy, K. A. (1994). Surviving the junior high school transition: Family processes and self-perceptions as protective and risk factors. *The Journal of Early Adolescence*, *14*(2), 162–199. 10.1177/027243169401400205.

[CR30] Lorenz, F., & Wild, E. (2007). Parental involvement in schooling: results concerning its structure and impact on students’ motivation. In M. Prenzel & L. Allolio-Näcke (Eds.), *Studies on the educational quality of schools. The final report on the DFG Priority Program* (299-316). Waxmann.

[CR31] Luis, T. M., Varela, R. E., & Moore, K. W. (2008). Parenting practices and childhood anxiety reporting in Mexican, Mexican American, and European American families. *Journal of Anxiety Disorders*, *22*, 1011–1020. 10.1016/j.janxdis.2007.11.001. 18083326 10.1016/j.janxdis.2007.11.001

[CR32] Marsh, H. W., & Parker, J. W. (1984). Determinants of student self-concept: Is it better to be a relatively large fish in a small pond even if you don’t learn to swim as well? *Journal of Personality and Social Psychology*, *47*(1), 213–231. 10.1037/0022-3514.47.1.213.

[CR33] Maslow, A. H. (1943). A theory of human motivation. *Psychological Review*, *50*(4), 370–396. 10.1037/h0054346.

[CR34] McCurdy, A. L., Williams, K. N., Lee, G. Y., Benito‐Gomez, M., & Fletcher, A. C. (2020). Measurement of parental autonomy support: A review of theoretical concerns and developmental considerations. *Journal of Family Theory & Review*, *12*(3), 382–397. 10.1111/jftr.12389.

[CR35] McLean, C. P. & Anderson, E. R. (2009). Brave men and timid women? A review of the gender differences in fear and anxiety. *Clinical Psychology Review*, *29*(6). 10.1016/j.cpr.2009.05.003. 10.1016/j.cpr.2009.05.003 19541399

[CR36] Muthén, L. K., & Muthén, B. O. (1998–2021). *Mplus User’s Guide* (8th ed.). Muthén & Muthén.

[CR37] Putwain, D., & Daly, A. L. (2014). Test anxiety prevalence and gender differences in a sample of English secondary school students. *Educational Studies*, *40*(5), 554–570. 10.1080/03055698.2014.953914.

[CR38] Putwain, D. W., Woods, K. A., & Symes, W. (2010). Personal and situational predictors of test anxiety of students in post compulsory education. *British Journal of Educational Psychology*, *80*(1), 137–160. 10.1348/000709909X466082. 19646333 10.1348/000709909X466082

[CR39] Ringeisen, T., & Raufelder, D. (2015). The interplay of parental support, parental pressure and test anxiety: Gender differences in adolescents. *Journal of Adolescence*, *45*(1), 67–79. 10.1016/j.adolescence.2015.08.018. 26378971 10.1016/j.adolescence.2015.08.018

[CR40] Ritchwood, T. D., Carthron, D., & Decoster, J. (2015). The impact of perceived teacher and parental pressure on adolescents’ study skills and reports of test anxiety. *Journal of Best Practices in Health Professions Diversity*, *8*(1), 1006–1019.

[CR41] Robson, D. A., Johnstone, J. S., Putwain, D. W., & Howard, S. (2023). Test anxiety in primary school children: A 20-year systematic review and meta-analysis. *Journal of School Psychology*, *98*, 39–60. 10.1016/j.jsp.2023.02.003. 37253582 10.1016/j.jsp.2023.02.003

[CR42] Rose, A. J., & Rudolph, K. D. (2006). A review of sex differences in peer relationship processes: Potential trade-offs for the emotional and behavioral development of girls and boys. *Psychol Bull*, *132*(1), 98–131. 10.1037/0033-2909.132.1.98. 16435959 10.1037/0033-2909.132.1.98 PMC3160171

[CR43] Rubin, K. H., Bukowski, W. M., & Bowker, J. C. (2015). Children in peer groups. In M. H. Bornstein, T. Leventhal, & R. M. Lerner (Eds.), *Handbook of child psychology and developmental science: Ecological settings and processes* (7th ed., pp. 175–222). John Wiley & Sons, Inc.

[CR44] Ryan, R. M., & Deci, E. L. (2000). Self-determination theory and the facilitation of intrinsic motivation, social development, and well-being. *American Psychologist*, *55*(1), 68–78. 10.1037/0003-066X.55.1.68. 11392867 10.1037//0003-066x.55.1.68

[CR45] Sebanc, A. M., Guimond, A. B., & Lutgen, J. (2016). Transactional relationships between latinos’ friendship quality and academic achievement during the transition to middle school. *The Journal of Early Adolescence*, *36*(1), 108–138. 10.1177/0272431614556347.

[CR46] Shadach, E., & Ganor-Miller, O. (2013). The role of perceived parental over-involvement in student test anxiety. *European Journal of Psychology of Education*, *28*(2), 585–596. 10.1007/s10212-012-0131-8.

[CR47] Sirsch, U. (2003). The impending transition from primary to secondary school: Challenge or threat? *International Journal of Behavioral Development*, *27*(5), 385–395. 10.1080/01650250344000082.

[CR48] Skinner, E. A., & Raine, K. E. (2023). Fostering the development of academic coping. In E. Skinner & M. Zimmer-Gembeck (Eds.), *The Cambridge Handbook of the Development of Coping* (Cambridge Handbooks in Psychology, pp. 641–679). Cambridge University Press.

[CR49] Skinner, E. A., & Wellborn, J. G. (1997). Children’s coping in the academic domain. In S. A. Wolchik & I. N. Sandler (Eds.), *Handbook of children’s coping: Linking theory and intervention* (pp. 387–422). Plenum Press. 10.1007/978-1-4757-2677-0_14.

[CR50] Song, J., Bong, M., Lee, K., & Kim, S.-I. (2015). Longitudinal investigation into the role of perceived social support in adolescents’ academic motivation and achievement. *Journal of Educational Psychology*, *107*(3), 821–841. 10.1037/edu0000016.

[CR51] Spinrad, T., Xu, X., Eisenberg, N., Dunbar, A., & Lozada, F. (2023). Parenting, socialization of emotion, and the development of coping. In E. Skinner & M. Zimmer-Gembeck (Eds.), *The Cambridge handbook of the development of coping* (Cambridge Handbooks in Psychology, pp. 447–467). Cambridge University Press. 10.1017/9781108917230.024.

[CR52] Symonds, J. E., & Galton, M. (2014). Moving to the next school at age 10–14 years: An international review of psychological development at school transition. *Review of Education*, *2*(1), 1–27. 10.1002/rev3.3021.

[CR53] Wang, J., Kaufman, T., & Branje, S. (2023). Longitudinal associations of parental psychological control and friend support with autonomy during early adolescence. *Journal of Research on Adolescence*, *33*(3), 999–1010. 10.1111/jora.12851. 37052955 10.1111/jora.12851

[CR54] Wells, C. S. (2021). Methods Based on Confirmatory Factor Analysis. In: *Assessing Measurement Invariance for Applied Research*. Educational and Psychological Testing in a Global Context. (pp. 295–368.). Cambridge University Press.

[CR55] Wigfield, A., & Eccles, J. S. (1989). Test anxiety in elementary and secondary school students. *Educational Psychologist*, *24*(2), 159–183. 10.1207/s15326985ep2402_3.

[CR56] Wigfield, A., Eccles, J. S., Fredricks, J. A., Simpkins, S., Roeser, R. W., & Schiefele, U. (2015). Development of achievement motivation and engagement. In M. E. Lamb & R. M. Lerner (Eds.), *Handbook of child psychology and developmental science: Socioemotional processes* (pp. 657–700). John Wiley & Sons, Inc. 10.1002/9781118963418.childpsy316.

[CR57] Wild, E. (1999). *Elterliche Erziehung und schulische Lernmotivation* [Parental education and school motivation]. Universität Mannheim.

[CR58] Valtin, R., & Wagner, C. (2004). *Der Übergang in die Sekundarstufe I: Psychische Kosten der externen Leistungsdifferenzierung* [Transition to secondary school: Psychological side-effects of external differentiation in school tracks]. *Psychologie in Erziehung und Unterricht*, *51*(1), 52–68.

[CR59] Vasquez, A. C., Patall, E. A., Fong, C. J., & Pine, L. (2016). Parent autonomy support, academic achievement, and psychosocial functioning: a meta-analysis of research. *Educational Psychological Review*, *28*, 605–644. 10.1007/s10648-015-9329-z.

[CR60] von der Embse, N., Jester, D., Roy, D., & Post, J. (2018). Test anxiety effects, predictors, and correlates: A 30-year meta-analytic review. *Journal of Affective Disorders*, *227*, 483–493. 10.1016/j.jad.2017.11.048. 29156362 10.1016/j.jad.2017.11.048

[CR61] Zeidner, M., & Matthews, G. (2003). Test anxiety. In R. Fernández-Ballesteros (Ed.). *Encyclopedia of Psychological Assessment* (pp. 965–969). SAGE Publications Ltd. 10.4135/9780857025753.n202.

[CR62] Zhang, Y., Watermann, R., & Daniel, A. (2023). The sustained effects of achievement goal profiles on school achievement across the transition to secondary school. *Journal of Youth Adolescence*, *52*, 2078–2094. 10.1007/s10964-023-01813-7. 37481504 10.1007/s10964-023-01813-7

[CR63] Zimmer-Gembeck, M. J., Gardner, A., & Kindermann, T. A. (2023). Peer stressors and peer relationship dynamics in the development of coping. In E. Skinner & M. Zimmer-Gembeck (Eds.), *The Cambridge Handbook of the Development of Coping* (Cambridge Handbooks in Psychology, pp. 538–559). Cambridge University Press. 10.1017/9781108917230.028.

[CR64] Zimmer-Gembeck, M. J., & Skinner, E. A. (2016). The development of coping: Implications for psychopathology and resilience. In D. Cicchetti (Ed.), *Developmental psychopathology: Risk, resilience, and intervention* (pp. 485–545). John Wiley & Sons, Inc. 10.1002/9781119125556.devpsy410.

