# Supplementary material for: Perceived Peer Integration, Parental Control, and Autonomy Support: Differential Effects on Test Anxiety during the Transition to Secondary School for Girls and Boys
Source: J Youth Adolesc. 2024 Jul 17;53(11):2610–22. doi: 10.1007/s10964-024-02053-z (PMC11467119; doi:10.1007/s10964-024-02053-z)
Supplement: Supplementary file 1 — Supplementary Material [file 10964_2024_2053_MOESM1_ESM.docx]

**Supplementary Material**

**Table 3**

*Confirmatory Factors Analyses for 1 vs. 2 Factor-Models of Test Anxiety in 4^th^-5^th^ Grade*

| Model |  | *χ²* | *df* | *p* | TLI | CFI | RMSEA | 90% CI | SRMR |
| --- | --- | --- | --- | --- | --- | --- | --- | --- | --- |
| Grade 4: 1-factor |  | 140.502 | 14 | <.001 | .960 | .940 | .073 | .063; .085 | .032 |
| Grade 4: 2-factor |  | 46.429 | 13 | <.001 | .983 | .989 | .039 | .027; .052 | .017 |
| Grade 5: 1-factor |  | 152.561 | 14 | <.001 | .927 | .951 | .078 | .067; .089 | .036 |
| Grade 5: 2-factor |  | 42.527 | 13 | <.001 | .983 | .990 | .037 | .025; .050 | .018 |

**Table 4**

*Measurement Invariance Testing for all Latent Variables*

| Model |  | *χ²* | *df* | *p* | TLI | CFI | ΔCFI | RMSEA | 90% CI | SRMR |
| --- | --- | --- | --- | --- | --- | --- | --- | --- | --- | --- |
| Worry^1^ | Longitudinal invariance |  |  |  |  |  |  |  |  |  |
|  | Configural | 16.288 | 5 | .006 | .994 | .982 | - | .036 | .017; 056 | .021 |
|  | Metric | 26.547 | 7 | <.001 | .990 | .978 | .004 | .040 | .024; .057 | .027 |
|  | Scalar | 48.349 | 9 | <.001 | .966 | .979 | -.001 | .050 | .037; .064 | .027 |
|  | Gender invariance |  |  |  |  |  |  |  |  |  |
|  | Metric 4^th^ grade | 0.158 | 2 | .924 | 1.007 | 1.000 | - | .000 | .000; .023 | .004 |
|  | Scalar 4^th^ grade | 17.143 | 4 | .002 | .975 | .983 | .017 | .063 | .034; .094 | .025 |
|  | Partial scalar 4^th^ grade | 0.166 | 3 | .983 | 1.007 | 1.000 | .000 | .000 | .000; .000 | .004 |
|  | Metric 5^th^ grade | 2.939 | 2 | .230 | .996 | .999 | - | .024 | .000; .078 | .019 |
|  | Scalar 5^th^ grade | 22.402 | 4 | <.001 | .959 | .973 | .026 | .075 | .047; .107 | .045 |
|  | Partial scalar 5^th^ grade | 4.973 | 3 | .174 | .994 | .997 | .002 | .028 | .000; .071 | .022 |
| EMO | Longitudinal invariance |  |  |  |  |  |  |  |  |  |
|  | Configural | 20.744 | 15 | .145 | .998 | .999 | - | .015 | .000; .029 | .014 |
|  | Metric | 27.880 | 18 | .064 | .996 | .997 | .002 | .018 | .000; .030 | .021 |
|  | Scalar | 30.349 | 21 | .085 | .997 | .997 | .000 | .016 | .000; .028 | .022 |
|  | Gender invariance |  |  |  |  |  |  |  |  |  |
|  | Configural: girls 4^th^ grade | 0.560 | 2 | .756 | 1.000 | 1.000 | - | .000 | .000; .046 | .003 |
|  | Configural: boys 4^th^ grade | 0.735 | 2 | .692 | 1.000 | 1.000 | - | .000 | .000; .052 | .005 |
|  | Configural 4^th^ grade | 1.307 | 4 | .860 | 1.006 | 1.000 | - | .000 | .000; .028 | .004 |
|  | Metric 4^th^ grade | 3.696 | 7 | .814 | 1.004 | 1.000 | .000 | .000 | .000; .026 | .015 |
|  | Scalar 4^th^ grade | 7.768 | 10 | .652 | 1.002 | 1.000 | .000 | .000 | .000; .031 | .021 |
|  | Configural: girls 5^th^ grade | 2.243 | 2 | .326 | .999 | 1.000 | - | .012 | .000; .071 | .008 |
|  | Configural: boys 5^th^ grade | 0.850 | 2 | .654 | 1.000 | 1.000 | - | .000 | .000; .055 | .005 |
|  | Configural 5^th^ grade | 3.129 | 4 | .536 | 1.002 | 1.000 | - | .000 | .000; .047 | .007 |
|  | Metric 5^th^ grade | 6.076 | 7 | .531 | 1.001 | 1.000 | .000 | .000 | .000; .040 | .017 |
|  | Scalar 5^th^ grade | 12.925 | 10 | .228 | .997 | .998 | .002 | .019 | .000; .045 | .016 |
| PI^1^ | Gender invariance |  |  |  |  |  |  |  |  |  |
|  | Metric 4^th^ grade | 3.449 | 2 | .178 | .991 | .997 | - | .030 | .000; .082 | .036 |
|  | Scalar 4^th^ grade | 8.410 | 4 | .078 | .986 | .991 | .006 | .037 | .000; .072 | .040 |
| PC | Gender invariance |  |  |  |  |  |  |  |  |  |
|  | Configural: girls 4^th^ grade | 4.290 | 2 | .117 | .984 | .995 | - | .036 | .000; .085 | .014 |
|  | Configural: boys 4^th^ grade | 6.033 | 2 | .049 | .977 | .992 | - | .050 | .003; .098 | .016 |
|  | Configural 4^th^ grade | 10.081 | 4 | .039 | .981 | .994 | - | .043 | .009; .076 | .015 |
|  | Metric 4^th^ grade | 19.028 | 7 | .008 | .979 | .988 | .006 | .045 | .021; .070 | .033 |
|  | Scalar 4^th^ grade | 24.829 | 10 | .006 | .982 | .985 | .003 | .042 | .021; .063 | .034 |
| PS | Gender invariance |  |  |  |  |  |  |  |  |  |
|  | Configural: girls 4^th^ grade | 0.660 | 2 | .720 | 1.010 | 1.000 | - | .000 | .000; .048 | .005 |
|  | Configural: boys 4^th^ grade | 1.277 | 2 | .528 | 1.006 | 1.000 | - | .000 | .000; .061 | .008 |
|  | Configural 4^th^ grade | 1.988 | 4 | .738 | 1.008 | 1.000 | - | .000 | .000; .037 | .007 |
|  | Metric 4^th^ grade | 5.429 | 7 | .608 | 1.004 | 1.000 | .000 | .000 | .000; .036 | .021 |
|  | Scalar 4^th^ grade | 7.436 | 10 | .684 | 1.004 | 1.000 | .000 | .000 | .000; .030 | .018 |

*Note.* Emo = Emotionality, PI = Peer Integration, PC = Parental Control, PS = Parental Autonomy Support.

^1^Due to just-identification, configural models could not be calculated in three-item measures (worry and peer integration).
